# Senescence-associated secretory phenotype in lung cancer: remodeling the tumor microenvironment for metastasis and immune suppression

**DOI:** 10.3389/fonc.2025.1605085

**Published:** 2025-05-29

**Authors:** Chen Chen, Ji Chen, Yanling Zhang, Qijun Zhang, Haixia Shi

**Affiliations:** ^1^ Department Traditional of Chinese Medicine (TCM), Shanghai Pudong New Area Pulmonary Hospital, Shanghai, China; ^2^ Department of Oncology, Integrated Traditional Chinese and Western Medicine Hospital of Shanghai Minhang District, Shanghai, China; ^3^ Department of Traditional Chinese Medicine, Shanghai Ninth People’s Hospital, Shanghai Jiao Tong University School of Medicine, Shanghai, China

**Keywords:** cellular senescence, lung cancer, SASP, therapy resistance, precision medicine

## Abstract

Cellular senescence exerts dual roles in lung cancer pathogenesis: initially suppressing tumorigenesis via p53/p21/p16-mediated cell cycle arrest, but promoting malignancy through the senescence-associated secretory phenotype (SASP). SASP secretes cytokines, proteases, and growth factors, reshaping the tumor microenvironment (TME) to drive immune evasion, metastasis, and therapy resistance. NF-κB activation induces APOBEC3B mutagenesis and PD-L1 overexpression, while mTOR signaling enhances glycolysis and OXPHOS to fuel tumor growth. Clinically, telomere attrition, p16/p21 expression, and SASP components serve as prognostic biomarkers. Therapeutic strategies target senescent cells and SASP. Future directions focus on single-cell multi-omics to decode senescence heterogeneity, spatially controlled drug delivery, and therapies targeting senescence-immune-metabolic crosstalk. By unraveling senescence’s dual regulatory mechanisms, this review highlights precision approaches to overcome resistance and improve lung cancer outcomes.

## 1 Introduction

Lung cancer remains the leading cause of global cancer mortality and the most prevalent malignant tumor, with adenocarcinoma constituting its predominant histological subtype (1). According to GLOBOCAN 2020 statistics, there were over 2.2 million new cases of lung cancer worldwide, accounting for 11.4% of all cancer cases. The number of deaths was approximately 1.79 million, representing 18% of the total cancer mortality. Among them, lung adenocarcinoma is the most common subtype of non-small cell lung cancer, accounting for about 40%–50% of NSCLC cases, with a higher incidence in women and non-smokers (2). As the most common form of non-small cell lung cancer (NSCLC), lung adenocarcinoma (LUAD) arises from malignant transformation of bronchial glandular cells, pathologically defined by glandular differentiation patterns and mucin-producing cellular architecture (3, 4). Distinct from other NSCLC subtypes, LUAD predominantly originates in peripheral lung structures including distal airways and alveoli, exhibiting characteristic histomorphological patterns such as acinar, papillary, micropapillary, and invasive mucinous adenocarcinoma (5). Clinical presentation often involves nonspecific respiratory symptoms—persistent cough, hemoptysis, dyspnea, and chest pain—frequently accompanied by constitutional manifestations like unexplained weight loss and fatigue (6). Its indolent early-stage progression explains why 20–30% of cases are incidentally detected through routine chest imaging (X-ray/CT), while over 60% present with locally advanced or metastatic disease at diagnosis (7). Epidemiologically, LUAD accounts for 40–50% of global lung cancer diagnoses, displaying unique demographic patterns: increased incidence in never-smokers, female predominance, higher prevalence in Asian populations, and elevated urban versus rural rates, potentially reflecting differential air pollution exposure (8). These epidemiological shifts, coupled with rising adenocarcinoma incidence rates, position LUAD as a critical driver of lung cancer’s persistent mortality burden (9). Despite the advancements in targeted therapies and immunotherapies that have significantly improved survival outcomes for some patients, the overall five-year survival rate for lung cancer remains below 20% (10). Particularly in advanced lung adenocarcinoma, issues such as drug resistance, recurrence, and immune evasion continue to pose significant challenges in clinical treatment, underscoring the urgent need to elucidate their molecular mechanisms and explore novel therapeutic strategies.

Molecular pathogenesis of LUAD centers on dysregulation of proliferative signaling cascades mediated by driver mutations (11). Epidermal growth factor receptor (EGFR) mutations represent the most prevalent oncogenic drivers, occurring in 10–50% of cases depending on population ethnicity and smoking status (12). Mutant EGFR acquires ligand-independent tyrosine kinase activity, constitutively activating downstream effectors including mitogen-activated protein kinase/extracellular signal-regulated kinase (MAPK/ERK, pro-survival signaling), phosphatidylinositol 3-kinase/protein kinase B (PI3K/AKT, anti-apoptotic signaling), and janus kinase/signal transducer and activator of transcription (JAK/STAT, proliferative/invasive regulation) pathways (13). Concurrently, PI3K/AKT/mTOR pathway hyperactivation promotes tumor metabolism and survival through enhanced glucose utilization and protein synthesis (14). Wnt/β-catenin signaling aberrations further contribute by sustaining cancer stem cell populations and apoptotic resistance (15). Moreover, kirsten rat sarcoma viral oncogene (KRAS) mutations, rearranged during transfection gene (RET) fusions, and ROS proto-oncogene 1 (ROS1) rearrangements represent pivotal oncogenic drivers in LUAD. KRAS mutations constitute the most prevalent driver mutation in EGFR-negative LUAD, notably with the smoking-associated KRAS G12C subtype predominating, accounting for around 13% of all NSCLC cases. By activating multiple downstream signaling pathways, including MAPK, PI3K/AKT, and RalGDS, KRAS mutations facilitate tumor proliferation, migration, and immune evasion (16). RET fusions predominantly occur in younger LUAD patients who are either non-smokers or light smokers, with an incidence rate of approximately 1–2%, and they are closely linked with accelerated tumor progression. RET fusions frequently result in fusion proteins with partner genes such as kinesin family member 5B (KIF5B) or coiled-coil domain containing 6 (CCDC6), thereby activating the MAPK and STAT pathways. As highly selective RET inhibitors, Selpercatinib and Pralsetinib have demonstrated remarkable objective response rates and favorable safety profiles in advanced RET fusion-positive LUAD, subsequently being incorporated into the recommended first-line treatment regimens (17). ROS1 fusion proteins propel tumor progression by engaging signaling pathways including PI3K/AKT and JAK/STAT. Crizotinib stands as the first approved targeted therapeutic for ROS1-rearranged NSCLC, demonstrating an efficacy rate exceeding 70% (18).

Similarly, epigenetic mechanisms, including DNA methylation, histone modifications and the dysregulation of non-coding RNAs (such as microRNAs and long non-coding RNAs), play pivotal roles in the initiation and progression of lung adenocarcinoma (19), while epigenetic dysregulation via abnormal DNA methylation, histone modifications, and non-coding RNA expression (miR-21 overexpression and lncRNA HOTAIR dysregulation) drives malignant transformation without altering genomic sequences (20).

The biological continuum of aging intersects critically with LUAD pathogenesis through cellular senescence mechanisms. Aging involves progressive functional decline across organ systems, mediated by hallmarks including genomic instability, telomere attrition, epigenetic drift, and mitochondrial dysfunction (21, 22). Cellular senescence—a permanent cell-cycle arrest triggered by oncogenic stress, DNA damage, or tumor suppressor activation—exerts context-dependent tumor-modulating effects (23). While initially tumor-suppressive by halting malignant transformation, senescent cells develop a senescence-associated secretory phenotype (SASP) (24), releasing inflammatory cytokines (IL-6, IL-8), growth factors (TGF-β), and proteases that remodel the tumor microenvironment (TME) (25). SASP components induce paracrine senescence in adjacent cells, recruit immunosuppressive myeloid cells, and paradoxically promote angiogenesis and metastasis through TME modulation (26, 27).

Emerging evidence positions senescence as a dual-axis regulator in LUAD progression (28). Lin et al. developed a 16-gene senescence-related signature (SRS) demonstrating that SASP-mediated immune microenvironment remodeling predicts immunotherapy response and survival outcomes (29). Complementary transcriptomic analyses of 278 senescence-associated genes revealed distinct senescence subtypes correlated with differential immune infiltration patterns in LUAD (30). These findings illuminate senescence as a dynamic interface between tumor biology and immune regulation, offering novel therapeutic targets—particularly for immunotherapy-resistant LUAD subtypes where SASP factors may mediate immune evasion. This review aims to systematically summarize the current understanding of cellular senescence in lung adenocarcinoma, with an emphasis on its dual roles in both tumor suppression and promotion. We particularly focus on the SASP and how it impacts tumor progression, immune modulation, and therapy resistance. Additionally, we discuss potential therapeutic opportunities and challenges in this context.

## 2 Association of cellular senescence with lung cancer

### 2.1 Telomere attrition

Telomere attrition serves as a critical nexus between cellular aging and lung carcinogenesis, driving chromosomal instability while paradoxically influencing tumor-suppressive and oncogenic pathways (31). Telomeres—terminal chromosomal regions composed of repetitive TTAGGG sequences and stabilized by shelterin protein complexes (TRF1/TRF2, TPP1)—prevent aberrant DNA repair by masking chromosomal ends from damage recognition systems (32). In somatic cells, the end-replication problem results in progressive telomere shortening (50–200 bp per division), culminating in replicative senescence when critical length thresholds (Hayflick limit) are breached; This triggers DNA damage response (DDR) activation through ataxia-telangiectasia mutated/ATM and rad3-related (ATM/ATR) kinases, stabilizing p53 to induce p21-mediated cell cycle arrest—a fundamental tumor-suppressive mechanism (33).

**Figure 1:**
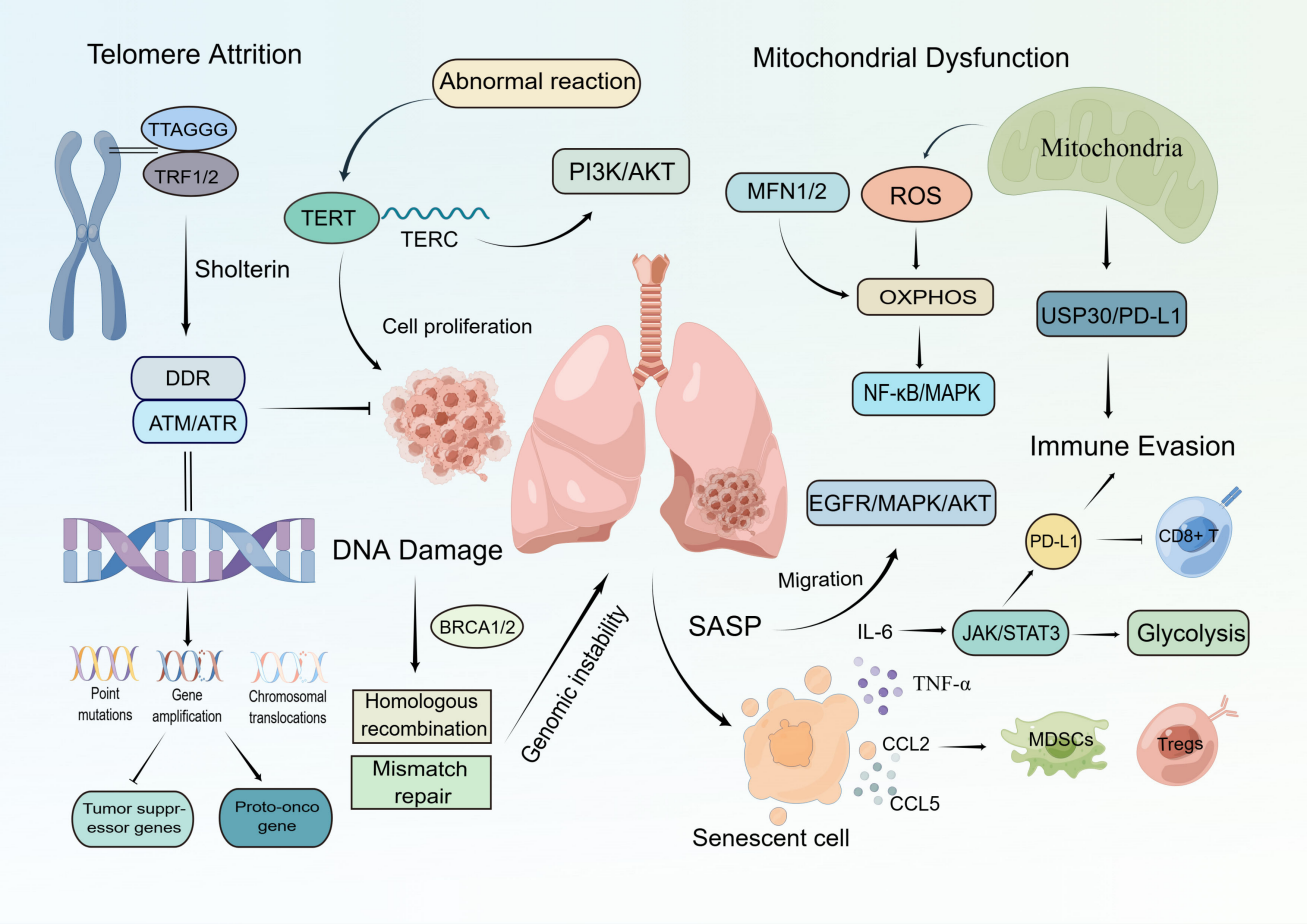
The Role of Cellular Senescence in Lung Cancer Progression. This figure illustrates the biological network through which cellular senescence drives lung cancer progression. (1)Telomere Attrition: Shortening of TTAGGG sequences and TRF1/2 imbalance disrupts Shelterin complex protection, activating ATM/ATR-mediated DDR. This induces genomic instability, leading to oncogene activation (EGFR, KRAS) and tumor suppressor inactivation (BRCA1/2); (2)DNA Damage: Defective homologous recombination repair (BRCA1/2) and mismatch repair (MMR) accelerate malignant clonal evolution; (3)Mitochondrial Dysfunction: MFN1/2 abnormalities elevate ROS, activating NF-κB/MAPK pathways to enhance cancer cell migration. Simultaneously, increased OXPHOS supports cancer stem cell survival; (4)Immune Evasion: ROS and PD-L1 suppress CD8^+^ T cell function, while USP30-mediated glycolytic reprogramming further weakens antitumor immunity; (5)Senescence-Associated Secretory Phenotype (SASP): Senescent cells secrete IL-6, TNF-α, and CCL2/5, recruiting MDSCs and Tregs to create an immunosuppressive microenvironment. This synergizes with EGFR/MAPK/AKT signaling to promote tumor growth and metastasis.

Contrasting this protective role, 85–90% of lung cancers exhibit pathological telomerase reactivation via telomerase reverse transcriptase (TERT, catalytic subunit) overexpression and telomerase RNA component (TERC, RNA template) dysregulation, enabling replicative immortality; Reactivation mechanisms include recurrent TERT promoter mutations (C228T/C250T) and epigenetic remodeling of telomere maintenance genes (34). Paradoxically, despite telomerase activity, lung tumors frequently display ongoing telomere attrition, generating chromosomal fusions and breakage-fusion-bridge cycles that amplify oncogenic signaling through PI3K/AKT and RAS-MAPK pathways while enhancing immune evasion via programmed death protein 1 (PD-L1)/PD-1 axis upregulation (35). Telomere attrition can elicit a persistent DNA damage response, thereby activating the ATM/ATR-checkpoint kinase 1 (CHK1) pathway and facilitating immune surveillance evasion by upregulating PD-L1 expression, ultimately promoting tumor cell immune tolerance and progression (36). Telomere disruption and the consequent loss of telomere-binding protein functionality can also activate cell survival signaling via the PI3K/AKT pathway, thereby enhancing tumor cell adaptability to oxidative stress and nutrient-poor conditions (37, 38). Clinically, leukocyte telomere length demonstrates bidirectional associations with lung adenocarcinoma risk: longer telomeres in peripheral blood correlate with heightened susceptibility, potentially reflecting inherited telomere maintenance defects or accelerated age-related shortening (39, 40). Mechanistically, while elongated telomeres can delay replicative senescence and extend the proliferative lifespan of somatic cells, they may also elevate the risk of accumulating genetic and epigenetic alterations under carcinogenic exposures, such as tobacco smoke, thereby increasing the potential for oncogenesis (41). Elongated telomeres are frequently accompanied by increased telomerase activity, which not only maintains chromosomal stability but also interacts with oncogenic pathways, such as MYC and TERT promoter mutations (42). This dual role positions telomere dynamics as both a biomarker and therapeutic target. Emerging strategies include TERT promoter inhibition using oligonucleotide antagonists such as GRN163L, telomerase splicing modulation through NOVA1-dependent alternative splicing blockade, and senescence-targeted therapies that combine senolytics (navitoclax) with SASP pathway inhibitors to mitigate pro-tumorigenic microenvironment effects, all of which represent promising approaches to advance lung cancer treatment (Shown in **Figure 1**) (43).

### 2.2 Carcinogenic effects of DNA damage and mutation accumulation

The accumulation of DNA damage and mutations constitutes a pivotal carcinogenic mechanism, driven by structural genomic alterations from endogenous sources—including replication errors and oxidative stress—and exogenous environmental carcinogens such as ultraviolet radiation and chemical agents (44).Unrepaired DNA lesions induce oncogenic transformation through point mutations, gene amplifications, or chromosomal translocations, which activate proto-oncogenes via gain-of-function mutations or inactivate tumor suppressors through loss of heterozygosity and epigenetic silencing (45). Compromised DNA repair pathways further amplify genomic instability: for example, breast cancer susceptibility gene 1/2(BRCA1/2) mutations disrupt homologous recombination (HR) repair, forcing reliance on error-prone mechanisms like non-homologous end joining, thereby accelerating oncogenic mutation accrual, while mismatch repair (MMR) deficiencies propagate microsatellite instability (46). Deficiency in HR repair serves as a principal driver of genomic instability across various tumor types. In LUAD, TP53 and KRAS mutations are intricately associated with defects in HR repair mechanisms. The loss of TP53 function compromises DNA damage checkpoint control, thereby synergistically promoting genomic instability and increased reliance on HR pathways. Conversely, KRAS mutations elevate ROS levels, thereby intensifying DNA damage stress and compelling tumor cells to rely more heavily on residual HR mechanisms for survival (47, 48). On the other hand, while MMR deficiencies are relatively uncommon in LUAD, their presence often results in a high tumor mutational burden (TMB), facilitating neoantigen formation and increasing sensitivity to immune checkpoint inhibitors (ICIs). The loss of function in core MMR genes such as muts homolog 2 (MSH2) and muts homolog 1 (MSH1) can lead to the upregulation of PD-L1 expression while activating the cyclic GMP-AMP synthase-stimulator of interferon genes (cGAS-STING) pathway, thereby inducing IFN-γ signaling to enhance tumor immunogenicity (49). PARP inhibitors obstruct the repair of single-strand breaks, thereby compelling cells to rely on the HR pathway to rectify DNA damage. Consequently, in tumor cells harboring BRCA1/2 mutations, where HRR function is compromised, the action of PARP inhibitors leads to the accumulation of DNA damage, triggering apoptosis—a phenomenon known as “synthetic lethality” (50).

Emerging approaches focus on replication stress mitigation using ATR/CHK1 inhibitors to bypass therapy resistance, alongside novel agents disrupting DNA damage tolerance pathways, collectively advancing precision oncology paradigms that capitalize on repair pathway dysregulation (51, 52). ATR and CHK1 are key kinases within the DDR network, primarily responding to replication stress and single-stranded DNA (ssDNA) damage. In LUAD, frequent mutations in genes such as TP53, KRAS, and ATM result in increased reliance of tumor cells on the ATR/CHK1 pathway (53). Ceralasertib (AZD6738), an ATR kinase inhibitor, markedly increases apoptosis, induces G2/M arrest, and enhances p21 expression while reducing CDC2 levels in SNU478 and SNU869 cell lines, demonstrating enhanced antitumor activity when combined with paclitaxel (54, 55). Prexasertib (LY2606368) is a substrate ATP competitive selective inhibitor of CHK1 and checkpoint kinase 2 (CHK2). In a phase I clinical trial involving patients with advanced squamous cell carcinoma, prexasertib monotherapy exhibited notable antitumor activity, with some patients achieving disease control after 3 months (56).

### 2.3 Mitochondrial dysfunction and cancer cell metabolism

Mitochondrial dysfunction drives cancer metabolic reprogramming by enabling survival advantages through energy metabolism remodeling, oxidative stress modulation, apoptosis evasion, and anabolic precursor synthesis (57). Although the Warburg effect historically dominated cancer metabolism paradigms, recent studies demonstrate that oxidative phosphorylation (OXPHOS) sustains the survival of therapy-resistant tumor subpopulations and metastatic cancer stem cells (58)—a phenomenon exemplified by glioblastoma stem cells that maintain immortality through mitochondrial fusion-mediated OXPHOS enhancement and NAD+ metabolic rewiring (59).This metabolic plasticity underpins therapeutic challenges, as lung cancers with elevated OXPHOS activity exhibit immunotherapy resistance, prompting the development of precision strategies like the OXPHOS inhibitor IACS-010759 to target refractory malignancies (60).

Mitochondrial reactive oxygen species (ROS) exhibit context-dependent oncogenic roles: mtDNA mutations or electron transport chain defects induce ROS overproduction, activating nuclear factor kappa-B (NF-κB) and MAPK pathways to drive lung cancer metastasis (61), while pharmacologic ROS modulation exerts antitumor effects. For instance, metformin suppresses ROS via complex I inhibition to sensitize tumors to chemotherapy, whereas pro-oxidant therapies exploit ROS overload to eliminate cancer stem cells (62). Parallel mechanisms involve mitochondrial regulation of apoptosis—overexpression of anti-apoptotic BCL-2 proteins (e.g., in lymphomas and breast cancers) blocks cytochrome c-mediated apoptosome activation, a vulnerability successfully targeted by the BCL-2 inhibitor venetoclax (63).

Mitochondrial metabolism plays a pivotal regulatory role in shaping the tumor immune microenvironment (TIME), significantly influencing immune evasion and antitumor immune responses (64). Tumor cells, by enhancing oxidative phosphorylation (OXPHOS) and aerobic glycolysis metabolism, accelerate nutrient consumption and produce lactate, leading to glucose and oxygen scarcity in TIME and the formation of an acidic microenvironment. This suppresses CD8^+^ effector T cell activity and promotes the expansion of regulatory T cells (Tregs) and myeloid-derived suppressor cells (MDSCs), creating an immunosuppressive niche (65). On the other hand, the proliferation and sustained functionality of effector and memory CD8^+^ T cells depend on mitochondrial OXPHOS and the tricarboxylic acid cycle (TCA). Mitochondrial dysfunction, such as loss of membrane potential and accumulation of reactive oxygen species (ROS), can lead to T cell exhaustion, closely related to the upregulation of immune checkpoint molecules like PD-1 (66). Therefore, targeting mitochondrial metabolism to remodel TIME and enhance T cell-mediated immune responses has become a key research direction in tumor immunotherapy. For instance, inhibiting pyruvate dehydrogenase kinase (PDK) can enhance TCA activity, promote acetyl-CoA production, and lead to increased histone acetylation, thereby boosting PD-L1 expression on tumor cells (67). In a triple-negative breast cancer (TNBC) mouse model, combined treatment with metformin and PD-1 antibodies significantly inhibited tumor growth and metastasis, increased CD8^+^ T cell infiltration, and reduced PD-L1 expression, indicating synergistic antitumor effects of the combination (68).

### 2.4 Promoting role of SASP in the carcinogenic microenvironment

The senescence-associated secretory phenotype (SASP) drives tumor progression through a multifaceted molecular network—comprising cytokines (IL-6, IL-8), chemokines (CXCL1, CCL2), proteases (MMPs), and growth factors (VEGF, TGF-β)—that remodels the tumor microenvironment (TME) into a pro-carcinogenic niche (69). SASP components directly amplify tumor proliferation and invasion: EREG/EGFR signaling activation via the MAPK/AKT axis mediates chemotherapy-induced progression in prostate cancer (70), while MMP1 and MMP3 degrade extracellular matrix (ECM) components to facilitate glioblastoma and lung cancer metastasis (71). Concurrently, SASP reprograms cancer metabolism; IL-6-induced STAT3 activation shifts energy production toward glycolysis while suppressing oxidative phosphorylation (OXPHOS), thereby fueling rapid tumor growth (72).

immune evasion (73). In KRAS-mutant lung cancer, senescent macrophages secrete CCL2 to recruit MDSCs, while IL-10 and TGF-β polarize tumor-associated macrophages (TAMs) toward an immunosuppressive M2 phenotype, crippling cytotoxic T cell activity (74). This immunosuppressive axis is reinforced by PD-L1 upregulation: IL-6 and VEGF activate PD-L1 expression on tumor cells, impairing NK cell function and CD8^+^ T cell-mediated cytotoxicity (75). Beyond immune modulation, SASP reshapes the stromal architecture by inducing fibrotic barriers. Cancer-associated fibroblasts (CAFs) secrete collagen and fibronectin under SASP influence, while lysyl oxidase (LOX)-mediated ECM crosslinking increases tissue stiffness, activating integrin-FAK signaling to accelerate metastasis and confer therapy resistance (76).

### 2.5 Regulation of immune evasion and inflammatory response by senescence

Cellular senescence orchestrates immune evasion and chronic inflammation in lung cancer through SASP-mediated immunosuppression and mitochondrial dysfunction (28).SASP-derived pro-inflammatory cytokines (IL-6, IL-8, TNF-α) and chemokines (CCL2, CXCL1) directly suppress antitumor immunity: IL-6 activates JAK/STAT3 signaling to upregulate PD-L1 expression on tumor cells and dendritic cells, inducing CD8^+^ T cell exhaustion (77). This mechanism corroborated by the 30% higher PD-L1 expression prevalence in elderly lung cancer patients compared to younger counterparts (78). Chemokine-driven immunosuppression is exemplified in KRAS-mutant lung cancer, where senescent cells recruit MDSCs and regulatory Tregs via CCL2 secretion, establishing an immune-privileged niche (79).

Senescent lung cancer cells further sabotage immune function through mitochondrial hijacking. Mutant mitochondria are transferred to T cells via tunneling nanotubes (TNTs), reducing T cell oxidative phosphorylation activity by 60% while activating the USP30-PD-L1 axis to amplify immune evasion—a process demonstrating direct crosstalk between metabolic dysfunction and checkpoint signaling (80). Concurrently, senescence-associated chronic inflammation fuels tumor progression through genomic destabilization: apolipoprotein B mRNA editing enzyme catalytic subunit 3B (APOBEC3B)-mediated cytosine deamination elevates mutation burden (2.5-fold higher in elderly patients) while NF-κB activation sustains pro-tumorigenic cytokine release (81). Therapeutic inhibition of NF-κB in aged preclinical models reduces lung tumor volume by 70%, underscoring the pathway’s centrality in senescence-driven malignancy (82). Collectively, these mechanisms drive immune surveillance failure and aggressive progression within the senescent lung cancer microenvironment (Shown in **Table 1**).

**Table 1:** Mechanisms of the impact of cellular senescence on lung cancer.

Senescence Mechanism	Mechanisms	Impact on Lung Cancer	Signaling Pathways/Molecules	Therapeutic Strategies
Telomere Attrition	Telomere attrition activates DNA damage response, inducing replicative senescence; telomerase reactivation bypasses senescence, enabling tumor immortality.	Promotes chromosomal instability; activates oncogenic pathways PI3K/AKT, RAS-MAPK; enhances immune evasion via PD-L1/PD-1 axis.	p53/p21; ATM/ATR; TERT promoter mutations	Telomerase inhibitors; TERT mutation-targeting drugs
DNA Damage and Mutation Accumulation	Endogenous/exogenous DNA damage leads to mutations in oncogenes or tumor suppressor inactivation.	Drives malignant transformation; induces APOBEC3B-mediated mutations;.	BRCA1/2; PARP; ATM/ATR	PARP inhibitors; immune checkpoint inhibitors; ATR/CHK1 inhibitors.
Mitochondrial Dysfunction	Impaired mitophagy causes ROS accumulation; OXPHOS supports therapy-resistant cancer stem cells; metabolic reprogramming.	Promotes metastasis; suppresses T cell function; enhances chemoresistance.	mTOR; HIF-1α; BCL-2 family	OXPHOS inhibitors; Metformin (glycolysis suppression); BCL-2 inhibitor.
SASP Secretion	SASP secretes IL-6, IL-8, MMPs and TGF-β, remodeling the tumor microenvironment.	Recruits MDSCs/Tregs to suppress immunity; promotes EMT and metastasis; activates EGFR-MAPK/AKT pathways.	NF-κB; STAT3; TGF-β/EGFR	SASP inhibitors; PD-1 inhibitor.
Chronic Inflammation and NF-κB	NF-κB activation drives SASP secretion, APOBEC3B mutations, and PD-L1 upregulation.	Enhances immune evasion; increases genomic instability.	NF-κB/APOBEC3B, PD-L1/PD-1	NF-κB inhibitors; anti-PD-L1 antibodies; BET inhibitors.
mTOR Pathway Dysregulation	mTORC1 promotes glycolysis and protein synthesis; mTORC2 activates EMT and metastasis.	Accelerates G1/S transition; drives chemoresistance.	PI3K/AKT/mTOR;RhoA/ROCK	mTOR inhibitors; MEK inhibitor; mTORC1-S6K1 inhibition.

## 3 Cellular senescence and lung cancer-related signaling pathways

### 3.1 Dual Roles of the p53/p21/p16 Signaling Pathway

The p53/p21/p16 axis exerts context-dependent tumor-suppressive and oncogenic effects in lung cancer through dynamic molecular crosstalk. Canonically, wild-type p53 activates p21 (cyclin dependent kinase Inhibitor 1A, CDKN1A) to enforce G1/S cell cycle arrest, enabling DNA repair or apoptosis initiation (83). This mechanism impaired in ~50% of lung cancers harboring TP53 mutations (84). Mutant p53 acquires oncogenic functions via epigenetic remodeling, including senescence-associated heterochromatin foci (SAHF) formation, which derepresses MYC transcription while silencing the cyclin dependent kinase Inhibitor 2A (CDKN2A) locus encoding p16 (85).

The p21 demonstrates paradoxical roles contingent on p53 status. In p53-wildtype tumors, p21-mediated CDK2/4 inhibition enhances chemotherapy response (86). Conversely, mutant p53 redirects p21 to upregulate RAD21, promoting homologous recombination repair and cisplatin resistance (87). Microenvironmental cues further modulate p21 activity: EGFR-STAT3 signaling phosphorylates p21 to induce its cytoplasmic translocation, where the p21/STAT3 complex activates AKT-mTOR signaling to drive metastasis (88).

The p16 (CDKN2A) inactivation—primarily through promoter hypermethylation—represents a hallmark of lung cancer progression, enabling cell cycle dysregulation via CDK4/6-RB pathway activation (89). Paradoxically, p16 loss upregulates telomerase (hTERT) to immortalize tumor cells, whereas its overexpression induces senescence, highlighting its dual regulatory capacity (90). Clinically, p16/p21 co-expression patterns predict immunotherapy efficacy: NSCLC patients with low p16 expression exhibit elevated PD-L1 levels but paradoxically inferior responses to PD-1 inhibitors, suggesting p16 loss primes an immune-evasive phenotype resistant to checkpoint blockade (91). These findings underscore the pathway’s complexity as a therapeutic determinant in lung cancer.

### 3.2 Cross-talk of NF-κB in lung cancer and cellular senescence

The NF-κB pathway functions as a molecular nexus linking cellular senescence to lung cancer progression through dual pro-survival and pro-inflammatory mechanisms. In senescent cells, DNA damage triggers ATM/ATR kinase activation, which phosphorylates the IKK complex to degrade IκBα, enabling nuclear translocation of the p65/p50 heterodimer—a prerequisite for SASP factor transcription (IL-6, IL-8, MMP9) that fosters a tumor-promoting inflammatory niche (92). Lung cancer cells amplify this cascade autonomously via TNF-α and HMGB1 secretion, creating a self-reinforcing NF-κB activation loop (93).

NF-κB-driven immune evasion operates through PD-L1 upregulation on tumor cells and stromal elements, suppressing CD8^+^ T cell cytotoxicity while recruiting myeloid-derived suppressor cells (MDSCs) and Tregs to establish an immunosuppressive barrier—a mechanism validated in therapy-resistant NSCLC subtypes (94). Concurrently, NF-κB exacerbates genomic instability by elevating APOBEC3B deaminase activity, inducing mutagenic C-to-T transitions in driver genes (EGFR, KRAS) that accelerate clonal evolution (95). Pro-inflammatory stimuli, such as TNF-α or IL-1β, activate NF-κB, which then binds to the promoter region of the APOBEC3B gene, resulting in increased transcriptional output. Elevated levels of APOBEC3B lead to widespread cytosine deamination in single-stranded DNA, contributing to a hypermutator phenotype and increased intratumoral heterogeneity. Recent studies have confirmed this axis in various cancers, including LUAD, linking chronic inflammation to tumor evolution through NF-κB-APOBEC3B-driven mutagenesis (96). Radiotherapy can induce nuclear translocation of NF-κB transcription factors, such as p65, by activating the ATM/IKK axis, thereby upregulating stemness genes like SOX2, NANOG, and ALDH1, and promoting self-renewal and survival of cancer stem cells (CSCs). Studies indicate that NF-κB activation is closely related to the enrichment of CSCs following radiotherapy, and its inhibition can significantly reverse CSC-associated phenotypes (97, 98). Furthermore, NF-κB drives the expression of pro-inflammatory factors such as IL-6, TNF-α, and CXCL1/2, promoting the infiltration of immunosuppressive cells like Tregs and MDSCs, and inducing upregulation of PD-L1. This culminates in the formation of TIME, weakening CD8^+^ T cell functionality and responsiveness to immunotherapy (99).

### 3.3 Regulation of the mTOR signaling pathway in cellular senescence and tumor development

The mTOR pathway functions as a metabolic integrator with dichotomous roles in cellular senescence and lung cancer progression, governed by its distinct complexes mTORC1 and mTORC2 (100). mTORC1 hyperactivation impairs mitophagy, causing accumulation of dysfunctional mitochondria and ROS overproduction, which drives p21-dependent senescence (101). Pharmacologic mTORC1 inhibition (rapamycin) reduces senescence-associated β-galactosidase (SA-β-Gal)-positive cells by 60% and rescues mitophagy, as demonstrated in *in vitro* senescence models (102). In contrast, mTORC2 exacerbates oxidative stress by suppressing SOD2 and catalase expression via AKT-mediated FOXO inactivation (103). Preclinical studies in lung preneoplasia show that mTORC2-selective inhibitor PP242 restores SOD2 levels to 80% of baseline and attenuates senescence-associated fibrosis by blocking FOXO3a phosphorylation (104). However, during lung cancer progression, aberrant activation of the mTOR pathway strongly promotes tumor malignancy (105). In established lung cancers, mTORC1 phosphorylates 4E-BP1/S6K1 to enhance ribosome biogenesis and oncoprotein synthesis (cyclin D1, c-MYC), accelerating G1/S progression (106). Clinically, 65% of lung tumors exhibit elevated p-S6K1 (indicating mTORC1 hyperactivity), correlating with poor prognosis (107). Studies show that mTORC2 is significantly activated in EGFR-mutant non-small cell lung cancer (NSCLC), and its functional upregulation is closely associated with tumor invasiveness, epithelial-mesenchymal transition (EMT), and TKI resistance (108). Furthermore, mTORC2 can enhance tumor cell metabolic adaptability by upregulating c-Myc and HIF-1α, thereby further promoting survival advantage under hypoxic conditions or treatment pressure (109). mTORC1 upregulates HIF-1α-dependent GLUT1 and LDHA to potentiate glycolysis, while mTORC2 enhances lipid biosynthesis via ACC activation, fulfilling anabolic demands of proliferating tumors (110, 111). These dual roles position mTOR as a context-dependent regulator: constraining senescence via metabolic homeostasis in pre-malignant states, yet driving malignancy through proliferative, invasive, and metabolic rewiring in advanced disease. In terms of treatment, although mTOR inhibitors such as rapamycin and its derivatives have entered clinical trials, they are mostly selective for mTORC1. Long-term use often induces feedback activation of the mTORC2-AKT pathway, limiting efficacy and potentially promoting resistance (112). Currently, rational combination strategies, such as pairing mTOR inhibitors with EGFR-TKI, PD-1/PD-L1 antibodies, or metabolic inhibitors, are considered key directions in enhancing efficacy and overcoming resistance (113).

## 4 Cellular senescence and lung cancer treatment

In lung cancer therapy, chemotherapy- or radiotherapy-induced senescent cells drive treatment resistance through senescence-associated secretory phenotype (SASP) activation. Senescent cells within the tumor microenvironment secrete pro-inflammatory cytokines, chemokines, and matrix remodeling enzymes, fostering chronic inflammation and immunosuppressive signaling (114). A key mechanism involves post-chemotherapy fibroblasts transferring zinc ions to cancer cells via the ZRT/IRT-like protein 1-connexin 43 (ZIP1-CX43) axis, which upregulates ABCB1-mediated drug efflux pumps to confer platinum resistance. ZIP1, as a zinc ion transporter, is extensively involved in maintaining intracellular zinc homeostasis, oxidative stress response, and metabolic regulation. Studies have shown that ZIP1 is underexpressed in various tumors, including prostate cancer and lung cancer, and is closely associated with metabolic reprogramming and apoptosis inhibition of tumor cells (115). Recently, studies focusing on the cooperative regulation between ZIP1 and gap junction protein CX43 have gained attention. ZIP1’s role in upregulating CX43 to form gap junctions between fibroblasts and lung cancer cells, facilitating zinc transfer and leading to chemotherapy resistance, has been highlighted (116). SASP factors further promote immune evasion: IL-6 enhances PD-L1 expression through STAT3 signaling to suppress CD8^+^ T cell activity, while CCL2 recruits MDSCs and regulatory Tregs, amplifying immunosuppression (116, 117). Senescent stromal cells exacerbate resistance by secreting serine peptidase inhibitor kazal type 1 (SPINK1), which activates the EGFR/STAT3 axis to inhibit apoptosis and stimulate metastasis (118). SPINK1 is a serine protease inhibitor that primarily inhibits trypsin activity under normal physiological conditions. Recent studies have shown that SPINK1 is abnormally overexpressed in prostate, pancreatic, and lung cancers, and is involved in regulating EGFR pathway activity, anti-apoptosis, and tumor stemness maintenance (119). In NSCLC, high SPINK1 expression is associated with poor prognosis in patients. SPINK1 promotes tumor cell growth and inhibits apoptosis by maintaining cellular redox homeostasis through activation of the nuclear factor erythroid 2-related factor 2 (NRF2) pathway; SPINK1 can also enhance migration and invasion capabilities of lung adenocarcinoma cells by upregulating the expression of matrix metalloproteinase 12 (120, 121). This interplay between SASP secretion, immune modulation, and metabolic remodeling underscores the critical role of senescent cells in driving therapeutic resistance and tumor progression in lung cancer.

Therapeutic elimination of senescent cells using senolytics has emerged as a strategy to overcome resistance (122). The BCL-2 inhibitor Navitoclax selectively targets chemotherapy-induced senescent lung cancer cells, demonstrating efficacy in preclinical models (123). In KRAS-mutant tumors, combining Navitoclax with PD-1 inhibitors elevates complete remission rates from 15% to 60% (124). Navitoclax has demonstrated the capability to eliminate senescent cells in clinical trials targeting idiopathic pulmonary fibrosis and myelofibrosis, such as NCT03289771 and NCT04592885 (125, 126). However, in oncological applications, the utility of Navitoclax is markedly constrained by dose-limiting thrombocytopenia (127). Similarly, Dasatinib-Quercetin co-treatment clears senescent fibroblasts by inhibiting SRC kinase and PI3K/AKT signaling, restoring T cell-mediated antitumor responses (128). The combined treatment strategy of Dasatinib-Quercetin has been validated in managing non-cancerous age-related conditions such as chronic kidney disease and osteoarthritis (NCT02848131) (129). In oncological models, Dasatinib-Quercetin has shown efficacy in eliminating chemotherapy-induced senescent cells and in retarding disease progression (130) However, in the domain of solid tumors, this approach remains in the early clinical stages, with long-term safety and efficacy requiring further evaluation.

SPINK1-neutralizing monoclonal antibodies block SASP-induced EGFR activation, synergizing with carboplatin to enhance cytotoxicity (118). In studies of hepatocellular carcinoma, SPINK1 neutralizing antibodies significantly downregulate VEGF and phosphorylated EGFR levels, thereby inhibiting tumor angiogenesis and the EMT process, subsequently delaying tumor progression (127). In a murine model of castration-resistant prostate cancer (CRPC), SPINK1 monoclonal antibodies markedly reduce the expression of neuroendocrine markers such as SYN and CHGA within tumors (131). CDK4/6 inhibitors, such as Palbociclib, Ribociclib, and Abemaciclib, have achieved significant advancements in the treatment of HR^+^/HER2^-^ breast cancer by blocking the cell cycle transition from G1 to S phase (132). However, tumor cells may circumvent CDK4/6 inhibition by upregulating the expression of CDK2, CCNE1, or E2F target genes, leading to treatment failure (133). Additionally, CDK4/6 inhibitors are metabolized via CYP3A4 and share metabolic pathways with various chemotherapeutic agents, which may result in abnormal drug plasma concentrations and increase the risk of adverse effects (134). Future directions include integrating single-cell metabolomics and spatial transcriptomics to map SASP regulatory networks (135), enabling precision strategies such as dual PD-1/SPINK1 checkpoint blockade or metabolic reprogramming with agents like metformin (136). Nevertheless, the field remains constrained by several technical challenges. Current mass spectrometry platforms often struggle with low metabolite abundance and limited dynamic range at the single-cell level, potentially compromising quantification accuracy (137). Moreover, the spatial and temporal resolution of metabolomic analysis remains insufficient, particularly in tissue contexts with complex microenvironments, such as lung tumors (137). Bioinformatics pipelines for integrating single-cell metabolomics data with transcriptomics or proteomics datasets are still under development, limiting interpretability (138). Addressing these bottlenecks is vital for realizing the full potential of SASP network analysis in mechanistic and clinical research.

## 5 Discussion

The dual roles of cellular senescence in lung cancer—acting as a tumor-suppressive mechanism via p53/p21/p16-mediated cell cycle arrest while driving malignancy through SASP-mediated inflammation—underscore its context-dependent impact on disease progression (139). SASP factors such as IL-6, IL-8, and MMPs activate oncogenic NF-κB and STAT3 signaling, with NF-κB upregulating APOBEC3B to induce EGFR/KRAS mutagenesis (140, 141), and STAT3 enhancing PD-L1 expression to suppress T cell cytotoxicity (142, 143). In NSCLC, SASP exhibits typical pro-inflammatory characteristics, primarily including factors such as IL-6, IL-8, CXCL1, and MMPs. These secretions can significantly enhance tumor invasiveness and heterogeneity by inducing EMT, promoting angiogenesis, and activating proliferative cancer-adjacent cells (144). Additionally, SASP can attract MDSCs and Tregs, shaping an immunosuppressive tumor microenvironment, thereby weakening the efficacy of immune checkpoint inhibitors (145). These mechanisms offer a therapeutic window for targeting SASP, especially in patients undergoing chemotherapy or radiotherapy that induces senescence, where senolytic drugs may help reduce recurrence and increase responses to immunotherapy (146). In contrast, SCLC is usually accompanied by the loss of p53 and Rb pathways, making it difficult for cells to enter the classic senescence program and hence lacking the typical SASP phenotype (147). Nevertheless, some studies indicate that SCLC can still exhibit atypical SASP-like phenotype post-treatment, with the released signaling factors potentially affecting tumor plasticity and cellular state transitions, such as neuroendocrine transdifferentiation (148). The high mutational burden of SCLC does not correlate with immunogenicity, possibly due in part to evasion of immune recognition by mechanisms such as downregulation of MHC-I, rather than relying on SASP’s constructed immunosuppressive network (149). Therefore, in SCLC, strategies targeting SASP have yet to show distinct clinical advantages, but the concept of inducing senescence or mimicking SASP to inhibit tumor activity still holds research potential.

Concurrently, mitochondrial dysfunction in senescent cells promotes metabolic reprogramming and ROS accumulation, fostering cancer stem cell survival and chemotherapy resistance. This is exacerbated by immunosenescence, exemplified by mitochondrial transfer to T cells via tunneling nanotubes, which cripples antitumor immunity and establishes a “metabolic-immune” barrier (150, 151). Senescence-associated biomarkers provide critical prognostic and therapeutic insights. Telomere length and TERT activity stratify immunotherapy responsiveness, with longer telomeres paradoxically correlating with poorer outcomes (152). Combined p16/p21 expression analysis predicts efficacy of immune checkpoint inhibitors (153), while dynamic monitoring of SASP factors (IL-6, CCL2) and APOBEC3B mutation burden—2.5-fold higher in elderly patients—guides synthetic lethality strategies like PARP inhibition (154). Single-cell sequencing has identified senescence-related gene signatures (senescence risk score, SRS) that enable molecular subtyping for precision therapy (155).

Therapeutic strategies targeting senescence focus on three pillars: senolytic elimination, SASP inhibition, and metabolic normalization. Navitoclax, a BCL-2 inhibitor, clears chemotherapy-induced senescent cells and synergizes with PD-1 inhibitors to boost tumor remission rates (156). JQ1, a BET inhibitor, epigenetically suppresses the IL-6/STAT3 axis to overcome EGFR-TKI resistance (157). Metformin reverses SASP-driven glycolysis and enhances T cell function, improving 5-year survival by 35% in diabetic lung cancer cohorts (158). Emerging approaches include DR5 agonist/cFLIP inhibitor combinations identified through multi-omics analysis and chronotherapy-optimized mTOR inhibitors to enhance CD8^+^ T cell activity (159). Despite significant progress in elucidating the role of senescence in lung adenocarcinoma, several challenges persist. Firstly, the heterogeneity and dynamic nature of senescent cells complicate the identification of universal markers or therapeutic targets. Secondly, the SASP demonstrates environment-dependent dual roles in tumor suppression and promotion, thereby complicating therapeutic modulation. Thirdly, the absence of reliable and specific senescence biomarkers in clinical lung cancer samples impedes effective patient stratification and comprehensive treatment monitoring. Finally, although senolytics and SASP inhibitors offer promising therapeutic avenues, their safety profiles, efficacy, and delivery mechanisms pose challenges, especially in combination therapies. Addressing these gaps remains critical for the successful translation of senescence-targeting strategies into effective clinical practice. Future research must address senescence heterogeneity and spatiotemporal dynamics. Spatial transcriptomics and metabolic flux analysis can map senescent cell niches, while AI-driven models integrating epigenetic, microbiome, and immune datasets may predict optimal therapeutic targets. Multidisciplinary innovations targeting the senescence-immune-metabolic axis will be pivotal in overcoming resistance and improving lung cancer survival.
